# Promising role of preoperative neutrophil-to-lymphocyte ratio in patients treated with radical nephroureterectomy

**DOI:** 10.1007/s00345-016-1848-9

**Published:** 2016-05-21

**Authors:** Mihai Dorin Vartolomei, Romain Mathieu, Vitaly Margulis, Jose A. Karam, Morgan Rouprêt, Ilaria Lucca, Aurélie Mbeutcha, Christian Seitz, Pierre I. Karakiewicz, Harun Fajkovic, Christopher G. Wood, Alon Z. Weizer, Jay D. Raman, Nathalie Rioux-Leclercq, Andrea Haitel, Karim Bensalah, Michael Rink, Alberto Briganti, Evanguelos Xylinas, Shahrokh F. Shariat

**Affiliations:** 1 Department of Urology, Vienna General Hospital, Medical University of Vienna, Vienna, Austria; 2 Department of Cell and Molecular Biology, University of Medicine and Pharmacy, Targu Mures, Romania; 3 Department of Urology, Rennes University Hospital, Rennes, France; 4 Department of Urology, University of Texas Southwestern Medical Center at Dallas, Dallas, TX USA; 5 Department of Urology, MD Anderson Cancer Center, Houston, TX USA; 6 Academic Department of Urology, La Pitié-Salpetrière Hospital, Assistance Publique-Hôpitaux de Paris, Faculté de Médecine Pierre et Marie Curie, University Paris 6, Paris, France; 7 Department of Urology, Centre hospitalier universitaire vaudois, Lausanne, Switzerland; 8 Cancer Prognostics and Health Outcomes Unit, University of Montreal Health Centre, Montreal, Canada; 9 Department of Urology, University of Michigan Cancer Center, Ann Arbor, MI USA; 10 Division of Urology, Penn State Milton S. Hershey Medical Center, Hershey, PA USA; 11 Department of Pathology, Rennes University Hospital, Rennes, France; 12 Department of Pathology, Medical University of Vienna, Vienna, Austria; 13 Department of Urology, University Medical Center Hamburg-Eppendorf, Hamburg, Germany; 14 Department of Urology, Vita Salute San Raffaele University, Milan, Italy; 15 Department of Urology, Cochin Hospital, Assistance Publique-Hôpitaux de Paris, Paris Descartes University, Paris, France; 16 Department of Urology, Weill Cornell Medical College, New York, NY USA; 17 Department of Urology, Comprehensive Cancer Center, Vienna General Hospital, Medical University of Vienna, Währinger Gürtel 18-20, 1090 Vienna, Austria

**Keywords:** Neutrophil-to-lymphocyte ratio, Prognostic factor, Urothelium, Carcinoma, Recurrence, Death

## Abstract

**Objective:**

Several retrospective studies with small cohorts reported neutrophil-to-lymphocyte ratio (NLR) as a prognostic marker in upper tract urothelial carcinoma (UTUC) following radical nephroureterectomy (RNU). We aimed at validating the predictive and prognostic role of NLR in a large multi-institutional cohort.

**Methods:**

Preoperative NLR was assessed in a multi-institutional cohort of 2477 patients with UTUC treated with RNU. Altered NLR was defined by a ratio >2.7. Logistic regression analyses were performed to assess the association between NLR and lymph node metastasis, muscle-invasive and non-organ-confined disease. The association of altered NLR with recurrence-free survival (RFS) and cancer-specific survival (CSS) was evaluated using Cox proportional hazards regression models.

**Results:**

Altered NLR was observed in 1428 (62.8 %) patients and associated with more advanced pathological tumor stage, lymph node metastasis, lymphovascular invasion, tumor necrosis and sessile tumor architecture. In a preoperative model that included age, gender, tumor location and architecture, NLR was an independent predictive factor for the presence of lymph node metastasis, muscle-invasive and non-organ-confined disease (*p* < 0.001). Within a median follow-up of 40 months (IQR 20–76 months), 548 (24.1 %) patients experienced disease recurrence and 453 patients (19.9 %) died from their cancer. Compared to patients with normal NLR, those with altered NLR had worse RFS (0.003) and CSS (*p* = 0.002). In multivariable analyses that adjusted for the effects of standard clinicopathologic features, altered NLR did not retain an independent value. In the subgroup of patients treated with lymphadenectomy in addition to RNU, NLR was independently associated with CSS (*p* = 0.03).

**Conclusion:**

In UTUC, preoperative NLR is associated with adverse clinicopathologic features and independently predicts features of biologically and clinically aggressive UTUC such as lymph node metastasis, muscle-invasive or non-organ-confined status. NLR may help better risk stratify patients with regard to lymphadenectomy and conservative therapy.

## Introduction

Upper tract urothelial carcinoma (UTUC) is a relatively rare disease with an annual incidence of 1–2 cases per 100,000 inhabitants [1]. Despite intense collaborative efforts to improve the knowledge of this disease, its management remains challenging [2]. The preoperative staging is therefore a major concern in UTUC since current imaging has still limited accuracy. Current predictive models based on preoperative parameters propose risk stratification on low-risk and high-risk tumors. These models may guide physicians for treatment decision making regarding the completion of a kidney-sparing procedure or a radical nephroureterectomy (RNU) with or without lymphadenectomy. RNU is indeed the standard treatment for high-risk UTUC. However, up to 30 % of the patients will experience early tumor recurrence after radical treatment and 80 % of these patients will eventually die from their disease [3, 4]. The prediction of oncologic outcomes is, therefore, another major concern in UTUC. The established prognostic models for UTUC mainly rely on definitive pathological features such as T stage, architecture, lymphovascular invasion, tumor location and concomitant in situ carcinoma [5]. However, these predictive and prognostic models need further optimization and further studies are needed to identify and validate new preoperative factors. Current efforts are focusing on biological and clinical biomarkers that capture the tumor behavior and reflect its intrinsic aggressiveness.

Markers of inflammatory response have been reported as potential biomarkers of tumor aggressiveness and worse outcome in several malignancies [6]. Indeed, tumor cells interact with their microenvironment and enhance local inflammation by releasing different cytokines and interleukins [7]. Such inflammatory setting could favor tumor progression while modifying levels of routine blood parameters such as C-reactive protein (CRP), leukocytes and derivatives. Several studies reported neutrophil-to-lymphocyte ratio (NLR), that combines neutrophils and lymphocytes, as a predictive factor for the presence of lymph node metastasis or non-organ-confined (NOC) disease in vulva squamous cell carcinoma [8] and in bladder cancer [9], respectively. Other studies demonstrated its prognostic value in pancreatic cancer, colon cancer or breast cancer [10]. In urological neoplasms, its prognostic value has been also reported in bladder cancer [11] and renal cell carcinoma [12]. In UTUC, evidence regarding its prognostic value is, however, limited to recent retrospective studies with relatively small cohorts [13–15], and none is known about its ability to predict adverse pathological features.

The aim of the present study was to externally validate the predictive and prognostic significance of pretreatment NLR in a large multi-institutional cohort from the UTUC collaboration.

## Patients and methods

### Patient selection and data collection

This study obtained an institutional review board approval in each institution, with all participating sites providing institutional data sharing agreements prior to the initiation of the study. 2477 patients treated with RNU for non-distant metastatic UTUC (Ta-T4N0-1M0) between March 1990 and May 2008 at institutions from the international UTUC collaboration were included. Patients with systemic diseases that could interfere with NLR at the time of RNU (such as leukemia, lymphoma, chronic inflammatory diseases, or autoimmune diseases), missing data or a follow-up <3 months (*n* = 203) were excluded. No patient received neoadjuvant chemotherapy.

### Study variables

Pretreatment NLR was assessed within the 30 days prior to RNU and defined as altered if ratio >2.7. This cutoff was based on previously published results [14]. Demographical, surgical, pathological, NLR (categorically coded according to cutoff) and outcomes data were collected and entered in a computerized database. Histology, tumor stage, grade, location, architecture, presence of lymphovascular invasion (LVI), tumor necrosis and carcinoma in situ (CIS) were confirmed by blinded re-review of the original pathology slides. The 2002 American Joint Committee on Cancer—Union International Centre le Cancer (AJCC-UICC) Tumor–Node–Metastasis (TNM) classification and the 1998 WHO/International Society of Urologic Pathology (ISUP) consensus classification were used for pathologic staging and grading, respectively.

### Management and follow-up

All patients had a standard RNU with bladder cuff removal with curative intent. A regional lymphadenectomy and adjuvant chemotherapy were completed at the discretion of the urologist. Follow-up was done according to institutional protocols in agreement with local guidelines at the time. Generally, patients were seen postoperatively quarterly for the first year, semiannually in the second year, and annually thereafter. Follow-up visits consisted of a physical examination, serum chemistry evaluation, urinary cytology and endoscopic examination of the bladder. A chest radiography and diagnostic imaging of the contralateral upper urinary tract, with a computerized tomography urogram, an ultrasonography and/or an intravenous pyelography, were performed annually. Chest computerized tomography and bone scan were performed at the discretion of the physicians. Recurrence was defined as any local recurrence (in the retroperitoneum or renal fossa) or distant metastasis. Recurrences in the bladder or contralateral upper urinary tract were considered as second primaries. Outcomes were measured by time to disease recurrence or to cancer-specific death. Cause of death was determined by the treating physician, based on chart review corroborated by death certificates, or by death certificates alone [16].

### Statistical analysis

Associations of NLR with categorical variables were assessed using Chi-square tests, while differences in continuous variables were analyzed using Kruskal–Wallis tests. Kaplan–Meier method was used to estimate recurrence-free survival (RFS) and cancer-specific survival (CSS); log-rank tests were applied for pair-wise comparison of survival. Muscle-invasive disease was defined as ≥pT2 and/or N+ disease, while NOC as ≥pT3 and/or N+ disease. Logistic regression analysis was performed to assess the association of NLR and other predictive factors with lymph node metastasis, muscle-invasive and NOC disease. Accuracy of the models was calculated using receiver operating characteristic (ROC) analysis. Univariable and multivariable Cox regression models addressed associations of RFS and CSS with potential prognostic factors. Subgroup analyses were done for patients with pTa-2 N0/Nx, pT1-3 N0/Nx, pT3/pT4 N0/Nx disease, high-grade tumors, positive and negative lymph node metastases or, treated with or without lymphadenectomy or adjuvant chemotherapy. All p values were two-sided, and statistical significance was defined as a *p* < 0.05. Statistical analyses were performed using Stata 11.0 statistical software (Stata Corp., College Station, TX, USA).

## Results

### Descriptive characteristics and association with altered NLR

Table 1 summarizes clinicopathologic characteristics of the cohort. A regional lymphadenectomy was performed in 729 patients (32.1 %). Adjuvant chemotherapy was administered in 217 patients (9.5 %). Altered NLR was observed in 1428 patients (62.8 %) and was associated with more advanced pathological tumor stage (*p* < 0.001), LVI (*p* < 0.001), tumor necrosis (*p* < 0.001), sessile architecture (*p* < 0.001) and lymph node metastases (*p* < 0.001) (Table 1).

**Table 1:** Association of neutrophil-to-lymphocyte ratio (NLR) and clinicopathologic characteristics in 2274 patients treated with radical nephroureterectomy for upper tract urothelial carcinoma

	All patients	Normal NLR	Altered NLR	*p*
Total, *n* (%)	2274	846 (37.2)	1428 (62.8)	
Age				0.66
Median (IQR)	69 (61–76)	69 (61–76)	70 (62–76)	
Gender, *n* (%)				0.31
Male	1527 (67.1)	579 (68.1)	948 (66.4)	
Female	747 (32.9)	267 (31.9)	480 (33.6)	
Tumor stage, *n* (%)				**<0.001**
pTa	497 (21.8)	199 (23.5)	298 (20.9)	
pTis	48 (2.2)	19 (2.2)	29 (2.3)	
pT1	532 (23.4)	244 (28.8)	288 (20.0)	
pT2	441 (19.4)	150 (17.8)	291 (20.3)	
pT3	671 (29.5)	217 (25.7)	454 (31.8)	
pT4	85 (3.7)	17 (2.0)	68 (4.7)	
Grade, *n* (%)				0.22
Low	367 (16.14)	147 (17.3)	220 (15.4)	
High	1907 (83.86)	699 (82.7)	1208 (84.6)	
Lymph node status, *n* (%)				**0.001**
pNx	1545 (68.0)	570 (67.4)	975 (68.2)	
pN0	545 (23.9)	228 (27.0)	317 (22.2)	
pN1	184 (8.1)	48 (5.6)	136 (9.6)	
Lymphovascular invasion, *n* (%)				**<0.001**
Yes	499 (21.9)	143 (16.9)	356 (24.9)	
No	1775 (78.1)	703 (83.1)	1072 (75.1)	
Concomitant carcinoma in situ, *n* (%)				0.17
Yes	528 (23.2)	183 (21.6)	345 (24.1)	
No	1746 (76.8)	663 (78.4)	1083 (75.9)	
Multifocality, *n* (%)				0.16
Yes	538 (23.7)	214 (25.3)	324 (22.7)	
No	1736 (76.3)	632 (74.7)	1104 (77.3)	
Necrosis, *n* (%)				**<0.001**
Yes	516 (22.7)	129 (15.2)	387 (27.1)	
No	1758 (77.3)	717 (84.8)	1041 (72.9)	
Architecture, *n* (%)				**0.001**
Papillary	1751 (77.0)	684 (80.9)	1067 (74.8)	
Sessile	523 (23.0)	162 (19.2)	361 (25.2)	
Location				0.67
Kidney	1448 (63.7)	534 (63.1)	914 (64.0)	
Ureter	826 (36.3)	312 (36.9)	514 (36.0)	

Statistically significant results are shown in bold

### Association of NLR with high-risk disease and lymph node metastasis

In univariable analysis, NLR was a predictive factor for lymph node metastasis, muscle-invasive and NOC disease (*p* ≤ 0.001, each) (Table 2). Similarly, kidney location and sessile architecture were independent predictors. In multivariable analysis that adjusted for the effects of age, gender, location and architecture, NLR retained an independent value for the prediction of all three pathologic features (*p* < 0.001, each). Addition of NLR improved by 2 points the accuracies of the models that predicted muscle-invasive and NOC disease (accuracy: 68 and 72 %, respectively) and 1 point the one dedicated to the prediction of the lymph node metastatic status (accuracy = 68 %).

**Table 2:** Univariable and multivariable logistic regression preoperative model including neutrophil-to-lymphocyte ratio for predicting lymph node metastases, muscle-invasive disease and non-organ-confined disease

Preoperative prognostic factors	Lymph node metastasis						pT3-4 or N+						≥T2 or N+					
	Univariable			Multivariable			Univariable			Multivariable			Univariable			Multivariable		
	HR	CI	*p*	HR	CI	*p*	HR	CI	*p*	HR	CI	*p*	HR	CI	*p*	HR	CI	*p*
NLR	1.74	1.24–2.46	**0.001**	1.2	1.14–2.29	**0.006**	1.51	1.26–1.82	**<0.001**	1.41	1.16–1.72	**0.001**	1.58	1.33–1.88	**<0.001**	1.49	1.24–1.80	**<0.001**
Age	0.99	0.98–1.00	0.50	0.99	0.97–1.00	0.26	1.00	0.99–1.01	0.08	1.00	0.99–1.01	0.33	1.00	0.99–1.01	0.17	1.00	0.99–1.01	0.68
Location	0.59	0.42–0.83	**0.003**	0.56	0.39–0.79	**0.001**	0.62	0.51–7.47	**<0.001**	0.52	0.42–0.64	**<0.001**	0.89	0.75–1.06	0.21	0.82	0.68–0.99	**0.05**
Gender	1.01	0.73–1.39	0.92	0.96	0.69–1.34	0.84	1.06	0.88–1.27	0.50	0.97	0.79–1.19	0.80	1.05	0.88–1.25	0.53	0.99	0.82–1.21	0.99
Architecture	3.40	2.50–4.62	**<0.001**	3.35	2.52–4.72	**<0.001**	6.78	5.47–8.40	**<0.001**	7.11	5.70–8.86	**<0.001**	8.45	6.46–11.06	**<0.001**	8.38	6.40–10.99	**<0.001**
Accuracy without NLR	**0.67**						**0.70**						**0.66**					
Accuracy with NLR	**0.68**						**0.72**						**0.68**					

Statistically significant results are shown in bold

*HR* hazard ratio, *CI* confidence interval, *p p* value, *NLR* neutrophil-to-lymphocyte ratio

### Association of NLR with cancer recurrence and cancer-specific survival

**Fig. 1:**
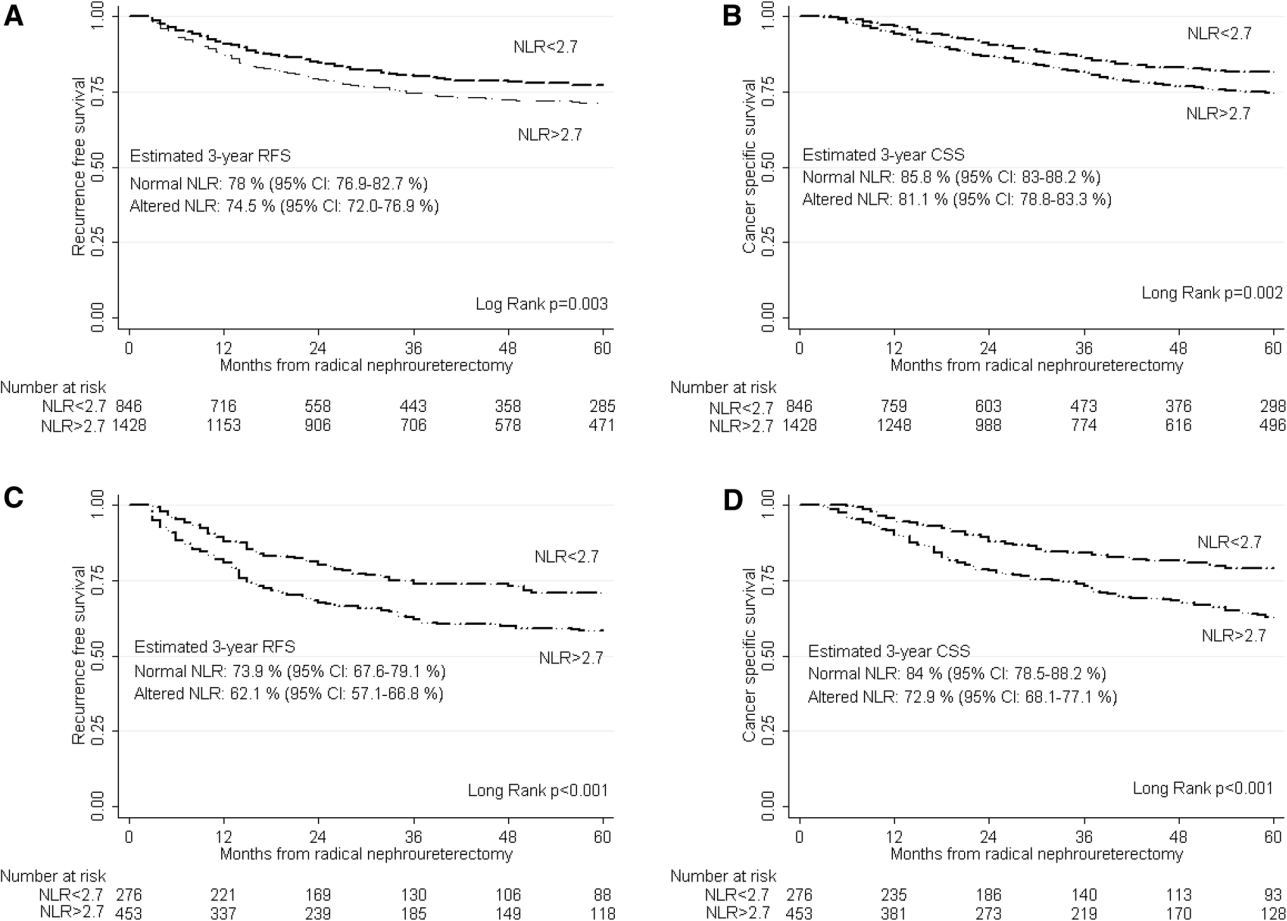
Kaplan–Meier estimates of recurrence-free survival and cancer-specific survival stratified by NLR status in 2274 patients with UTUC treated with RNU alone (**a**, **b**, respectively), and 729 patients with UTUC treated with RNU and lymphadenectomy (**c**, **d**, respectively). *RFS* recurrence-free survival, *CSS* cancer-specific survival, *CI* confidence interval, *NLR* neutrophil-to-lymphocyte ratio, *UTUC* upper tract urothelial carcinoma, *RNU* radical nephroureterectomy

Within a median follow-up of 40 months (range 20–76 months), 548 patients (24.1 %) experienced disease recurrence and 453 patients (19.9 %) died from their cancer. Patients with altered NLR had worse RFS and CSS than those with normal NLR (*p* < 0.003 and *p* = 0.002, respectively). 3-year RFS and CSS estimates were: 78 (CI 76.9–82.7 %) and 85.8 % (CI 83–88.2 %) for patients with normal NLR, and 74.5 (CI 72.0–76.9 %) and 81.1 % (CI 78.8–83.3 %) for patients with abnormal NLR, respectively (Fig. 1a, b).

Table 3 shows the univariable and multivariable Cox proportional hazard regression analyses in the overall cohort. In univariable analysis, altered NLR was significantly associated with RFS and CSS (HR = 1.30; *p* = 0.003 and HR = 1.36; *p* = 0.002, respectively); however, when adjusted for the effects of clinicopathologic features, NLR did not retain its statistical significance for both endpoints (HR = 1.05; *p* = 0.59 and HR = 1.07; *p* = 0.48, respectively). Similar results were observed in all subgroup analyses performed according to T stage, grade, lymph node status or adjuvant chemotherapy (Table 4).

**Table 3:** Univariable and multivariable Cox regression analyses predicting recurrence and cancer-specific mortality of 2274 patients treated with radical nephroureterectomy for upper tract urothelial carcinoma

	Recurrence-free survival						Cancer-specific survival					
	Univariable			Multivariable			Univariable			Multivariable		
	HR	95 % CI	*p* value	HR	95 % CI	*p* value	HR	95 % CI	*p* value	HR	95 % CI	*p* value
Age	1.01	1.00–1.02	<0.001	1.01	1.00–1.02	0.001	1.02	1.01–1.03	<0.001	1.02	1.01–1.03	<0.001
Gender	1.10	0.92–1.31	0.27	1.01	0.84–1.21	0.89	1.06	0.87–1.29	0.52	0.94	0.77–1.15	0.58
Stage												
Ta	Ref.											
Tis	2.98	1.30–6.83	0.01	1.61	0.94–7.25	0.066	3.58	1.44–8.88	0.006	3.37	1.08–10.56	0.03
T1	2.16	1.38–3.37	<0.001	1.87	0.89–3.91	0.095	2.00	1.18–3.37	0.009	1.66	0.70–3.92	0.24
T2	5.01	3.30–7.59	<0.001	3.84	1.86–7.92	<0.001	5.54	3.45–8.91	<0.001	4.11	1.79–9.45	<0.001
T3	10.29	6.97–15.19	<0.001	6.96	3.40–14.21	<0.001	11.93	7.63–18.65	<0.001	7.85	3.45–17.88	<0.001
T4	39.75	25.36–62.29	<0.001	19.94	9.26–42.96	<0.001	46.14	27.74–76.75	<0.001	21.30	8.86–51.22	<0.001
Grade	6.17	3.94–9.64	<0.001	1.10	0.48–2.50	0.81	6.93	4.14–11.60	<0.001	1.17	0.45–3.03	0.73
Lymphovascular invasion	3.13	2.64–3.71	<0.001	1.24	1.02–1.52	0.029	3.39	2.81–4.09	<0.001	1.35	1.08–1.67	0.006
Architecture	3.30	2.78–3.91	<0.001	1.37	1.12–1.66	0.002	3.46	2.87–4.17	<0.001	1.36	1.10–1.69	0.004
Carcinoma in situ	1.67	1.39–2.00	<0.001	1.23	1.01–1.50	0.035	1.57	1.28–1.92	<0.001	1.08	0.87–1.35	0.46
Necrosis	2.22	1.86–2.64	<0.001	0.98	0.81–1.19	0.90	2.25	1.86–2.74	<0.001	1.00	0.81–1.23	0.98
Multifocality	1.31	1.08–1.58	0.004	0.91	0.74–1.11	0.36	1.33	1.08–1.64	0.006	0.95	0.76–1.70	0.66
Location	1.11	0.94–1.32	0.19	1.31	1.09–1.57	0.003	1.16	0.96–1.40	0.11	1.39	1.14–1.70	<0.001
Lymph node metastases Nx	Ref.											
N0	1.13	0.92–1.40	0.22	0.91	0.73–1.12	0.39	0.99	0.77–1.26	0.94	0.77	0.60–0.98	0.04
N1	5.26	4.25–6.51	<0.001	2.10	1.64–2.68	<0.001	5.24	4.16–6.59	<0.001	1.96	1.51–2.56	<0.001
NLR	1.30	1.09–1.56	0.003	1.05	0.87–1.26	0.59	1.36	1.11–1.66	0.002	1.07	0.87–1.31	0.48

*CI* confidence interval, *HR* hazard ratio, *NLR* neutrophil-to-lymphocyte ratio

**Table 4:** Univariable and multivariable Cox regression analyses for prediction of recurrence and cancer-specific mortality according NLR status in subgroups of patients treated with radical nephroureterectomy

Patient subgroups	Recurrence-free survival						Cancer-specific survival					
	Univariable			Multivariable			Univariable			Multivariable		
	HR	95 % CI	*p* value	HR	95 % CI	*p* value	HR	95 % CI	*p* value	HR	95 % CI	*p* value
pTa-4 high grade	1.28	1.07–1.54	0.007	1.05	0.87–1.26	0.65	1.34	1.09–1.64	0.005	1.06	0.87–1.31	0.54
pTa-2 pN0/Nx	0.98	0.74–1.31	0.91	0.85	0.63–1.13	0.27	1.02	0.73–1.41	0.92	0.86	0.62–1.20	0.38
pT1-3 pN0/Nx	1.07	0.87–1.33	0.51	0.92	0.74–1.14	0.43	1.20	0.94–1.52	0.14	1.01	0.79–1.29	0.92
pT3/pT4 N0/Nx	1.16	0.87–1.53	0.31	1.12	0.84–1.49	0.44	1.26	0.92–1.72	0.1	1.20	0.87–1.64	0.27
pT2-4 pN0/Nx	1.08	0.86–1.36	0.51	1.01	0.80–1.28	0.90	1.11	0.86–1.43	0.40	1.03	0.80–1.33	0.82
pTa-4 pN0	1.30	0.90–1.89	0.16	1.17	0.79–1.74	0.42	1.69	1.07–2.67	0.02	1.47	0.91–2.38	0.1
pTa-4 pN1	1.54	0.99–2.39	0.05	1.30	0.81–2.09	0.27	1.39	0.88–2.19	0.15	1.20	0.73–1.98	0.46

*CI* confidence interval, *HR* hazard ratio

### Association of NLR with cancer recurrence and cancer-specific survival after RNU with lymphadenectomy

The median number of lymph nodes removed during lymphadenectomy was 5 (IQR 2–10). Among the patients treated with lymphadenectomy, 239 (32.8 %) experienced tumor recurrence and 192 (26.3 %) died from the disease. NLR was altered in 170 (71.1 %) and 141 (73.4 %) patients, respectively. On Kaplan–Mayer analysis, altered NLR was associated with both worse RFS and CSS (*p* < 0.001, each). 3-year RFS and CSS were 62.1 and 72.9 % in patients with altered NLR, compared to 73.9 and 84 % in patients with normal NLR (Fig. 1c, d). NLR was associated with RFS (HR 1.63; IC 1.23–2.16, *p* < 0.001) and CSS (HR 1.79; IC 1.30–2.47, *p* < 0.001) on univariable analyses. When adjusted for the effects of clinicopathologic features, NLR retained its statistical significant association with CSS (HR 1.43; IC 1.02–2.00, *p* = 0.03), but not with RFS (HR 1.29; IC 0.96–1.73, *p* = 0.08) (Table 5).

**Table 5:** Univariable and multivariable Cox regression analyses predicting the recurrence and cancer-specific mortality of 729 patients treated with radical nephroureterectomy and lymphadenectomy for upper tract urothelial carcinoma

	Recurrence-free survival						Cancer-specific survival					
	Univariable			Multivariable			Univariable			Multivariable		
	HR	95 % CI	*p* value	HR	95 % CI	*p* value	HR	95 % CI	*p* value	HR	95 % CI	*p* value
Age	1.01	1.00–1.02	0.01	1.01	0.99–1.02	0.06	1.02	1.00–1.03	<0.001	1.02	1.00–1.03	**0.005**
Gender	1.23	0.95–1.60	0.11	1.14	0.86–1.49	0.34	1.06	0.79–1.43	0.66	0.93	0.68–1.27	0.67
Stage												
Ta	Ref.											
Tis	1.06	0.12–8.87	0.95	1.47	0.13–16.43	0.75	1.52	0.17–13.06	0.70	3.81	0.23–61.67	0.34
T1	2.34	0.93–5.90	0.07	2.76	0.63–11.98	0.17	1.41	0.47–4.23	0.53	2.63	0.33–20.90	0.36
T2	5.24	2.23–12.31	<0.001	5.37	1.28–22.42	**0.02**	4.34	1.69–11.13	0.002	6.64	0.89–49.14	0.06
T3	10.22	4.50–23.17	<0.001	8.68	2.12–35.50	**0.003**	10.03	4.09–24.61	<0.001	12.34	1.70–84.46	**0.01**
T4	32.13	13.57–76.07	<0.001	19.95	4.66–85.29	**<0.001**	33.0	12.90–84.60	<0.001	25.92	3.45–194.75	**0.002**
Grade	6.76	2.51–18.18	<0.001	0.73	0.13–4.04	0.72	5.30	1.97–14.29	<0.001	0.36	0.04–3.26	0.36
Lymphovascular invasion	2.45	1.90–3.16	<0.001	0.88	0.66–1.18	0.42	2.92	2.19–3.88	<0.001	1.05	0.76–1.45	0.74
Architecture	3.05	2.36–3.94	<0.001	1.48	1.10–2.00	**0.009**	3.39	2.55–4.52	<0.001	1.58	1.13–2.20	**0.007**
Carcinoma in situ	1.38	1.06–1.79	0.01	1.17	0.88–1.56	0.26	1.31	0.97–1.77	0.07	0.94	0.68–1.31	0.74
Necrosis	1.97	1.52–2.54	<0.001	1.02	0.77–1.35	0.85	1.97	1.48–2.62	<0.001	1.02	0.75–1.39	0.86
Multifocality	1.12	0.84–1.48	0.42	0.82	0.60–1.11	0.20	1.32	0.97–1.80	0.07	0.99	0.71–1.39	0.98
Location	0.97	0.74–1.27	0.87	1.23	0.93–1.64	0.13	1.02	0.76–1.37	0.87	1.31	0.95–1.79	0.09
Lymph node metastases	4.61	3.57–5.96	<0.001	2.62	1.95–3.52	**<0.001**	5.23	3.93–6.97	<0.001	2.75	1.98–3.83	**<0.001**
NLR	1.63	1.23–2.16	<0.001	1.29	0.96–1.73	0.08	1.79	1.30–2.47	<0.001	1.43	1.02–2.00	**0.03**

Statistically significant results are shown in bold

*CI* confidence interval, *HR* hazard ratio, *NLR* neutrophil-to-lymphocyte ratio

## Discussion

In the present study, we assessed the significance of preoperative NLR in a large multi-institutional cohort of patients with UTUC treated by RNU. We demonstrated that abnormal NLR was not only associated with adverse pathological features and worse oncologic outcomes, but also predicted the presence of lymph node metastases, muscle-invasive and NOC disease. Therefore, the potential of NLR could be in the preoperative clinical decision making regarding lymphadenectomy indication and extent, and patient counseling regarding conservative therapy.

Using a ratio of 2.7, we observed that almost two-thirds of the patients had an altered preoperative NLR. Altered NLR was associated with features of biologically and clinically aggressive UTUC such as advanced tumor stage, LVI, tumor necrosis, sessile architecture and lymph node metastases. These findings confirm the results reported in smaller cohorts [13–15]. The potential reasons for such associations remain hypothetical. NLR could reflect a balance between neutrophilia and lymphopenia that results from the relationship between the immune system and tumorigenesis. On the one hand, the secretion of granulocyte colony-stimulating factor (G-CSF) by tumor cells may promote neutrophils production in bone marrow and recruitment of the neutrophils in the tumor environment. De Larco et al. [7] showed that these “tumor-associated neutrophils” could have a role in the tumor microenvironment and local angiogenesis. Neoangiogenesis could therefore promote tumor progression and migration of tumor cells. On the other hand, lymphopenia may be responsible for poor immune response against tumor and favor tumor aggressiveness and progression [17]. Even if this association is of great interest, it does not ensure the independent value of NLR to predict adverse pathological features at final pathology.

We demonstrated, therefore, the ability of NLR to independently predict lymph node metastasis, muscle-invasive and NOC disease in a preoperative model that included patient age, gender, tumor location and architecture. This preoperative model may help physicians identify patients who should be proposed a radical treatment with RNU or a lymphadenectomy during RNU [18]. Previous models have been proposed to identify such patients and adapt clinical decision making [19–21]. Margulis et al. [19] developed a model to predict muscle-invasive disease using preoperative clinicopathologic features such as age, gender, tumor location, architecture and grade at biopsy. Another predictive model combined high grade, tumor location, local invasion and hydronephrosis on imaging and achieved 71 and 70 % accuracies for predicting muscle-invasive and NOC disease, respectively [20]. We constructed a model including NLR but did not consider, however, grade at biopsy and data from the preoperative imaging. Our model only relies on easy accessible factors and demonstrates accuracies of 68–72.5 % for the prediction of NOC, muscle-invasive or positive lymph node disease. Further improvement of these models may be obtained, however, by the combination of NLR with other routinely available biomarkers as CRP [22].

We demonstrated NLR was associated with worse oncologic outcomes such as RFS and CSS. However, adjusted for standard pathologic prognostic factors in UTUC, NLR did not retain statistical significance. Most of the previous studies reported, however, independent predictive value for RFS [15], CSS [13, 14] and OS [14]. The discrepancies between these studies and our findings could be explained by several reasons. First, initial studies defined the optimal cutoff for NLR as 2.7 [14] or 3 [13] based on the interpretations of iterative Cox or ROC analysis observed in the cohorts. Only one study externally validated the prognostic value of NLR using a predefined threshold of 3 [15]. To our knowledge, we are the first to propose an external validation with the 2.7 cutoff proposed by Dalpiaz et al. [14]. Second, the parameters used in multivariable analysis proposed in these studies varied widely. For example, Tanaka et al. only adjusted on age, N status, stage and LVI. We performed a multivariable analysis using most of the clinicopathologic parameters published in the literature [3, 23, 24]. When we limited our multivariable analysis to the parameters used in established prognostic models [3, 23–25], NLR did not demonstrate independent prognostic value, however (data not shown). Third, populations were different regarding the clinicopathologic characteristics. Luo et al. [13] only included patients with pT stage <4 and without lymph node metastases. Conversely, half cohort of Tanaka et al. [15] had locally advanced disease. However, none of the subgroup analyses we performed with advanced disease or localized disease demonstrated independent prognostic value for NLR.

We demonstrated, however, that NLR would be an independent prognostic factor regarding CSS in patients who were treated with lymphadenectomy. In previous studies, status of lymphadenectomy was not routinely performed [13–15]. Staging benefit of lymphadenectomy in UTUC is noteworthy [26]. Xylinas et al. [27] showed, therefore, that all patients treated with RNU for UTUC should receive LND to ensure accurate nodal staging. In the future, metastatic patients may also receive immune checkpoint inhibitors such as anti-PDL1. Preliminary results in bladder cancer suggest that circulating inflammatory markers may predict response to immune checkpoint inhibitors [28]. Therefore, NLR could be of great value in the identification of patients with metastatic status that may benefit from immune checkpoint inhibitors.

Although this is the largest study that investigated the predictive and prognostic value of pretreatment NLR with outcomes after RNU, our study has some limitations that should be considered. First, its retrospective and multicentric status may be responsible for variations in laboratory, pathological and surgical workup that could confound the results. In the present study, NLR was determined preoperatively with a predefined cutoff and analyzed as a categorical variable. We did not complete these investigations with exact levels of NLR as neutrophils and lymphocytes counts were measured with different assays. Continuously coded NLR or different thresholds may have provided different conclusions. Assessment of NLR at different points in time may also have excluded confounders, such as occult infectious diseases without any symptoms preoperative, autoimmune diseases responsible for temporally changes. Finally, we only investigated NLR. A growing evidence suggests that derivatives of NLR or its combination with other preoperative markers of systemic inflammation may be helpful in the prediction of oncologic outcomes in UTUC and warrants further investigations [29–31].

## Conclusion

In UTUC, NLR is associated with adverse clinicopathologic features and worse oncologic outcomes. However, its prognostic role may be limited to patients treated with lymphadenectomy. Conversely, all patients could benefit in the preoperative setting from its ability to independently predict lymph node metastasis, muscle-invasive and NOC disease. NLR may be therefore a useful biomarker in clinical decision making regarding a radical treatment and the completion and extent of a lymphadenectomy.
